# Current Applications and Future Directions of Bioengineering Approaches for Bladder Augmentation and Reconstruction

**DOI:** 10.3389/fsurg.2021.664404

**Published:** 2021-06-18

**Authors:** Xuesheng Wang, Fan Zhang, Limin Liao

**Affiliations:** ^1^Department of Urology, China Rehabilitation Research Center, Rehabilitation School of Capital Medical University, Beijing, China; ^2^Department of Urology, Capital Medical University, Beijing, China; ^3^University of Rehabilitation, Qingdao, China

**Keywords:** bladder augmentation, scaffolds, tissue engineering, 3D bioprinting, bladder reconstruction

## Abstract

End-stage neurogenic bladder usually results in the insufficiency of upper urinary tract, requiring bladder augmentation with intestinal tissue. To avoid complications of augmentation cystoplasty, tissue-engineering technique could offer a new approach to bladder reconstruction. This work reviews the current state of bioengineering progress and barriers in bladder augmentation or reconstruction and proposes an innovative method to address the obstacles of bladder augmentation. The ideal tissue-engineered bladder has the characteristics of high biocompatibility, compliance, and specialized urothelium to protect the upper urinary tract and prevent extravasation of urine. Despite that many reports have demonstrated that bioengineered bladder possessed a similar structure to native bladder, few large animal experiments, and clinical applications have been performed successfully. The lack of satisfactory outcomes over the past decades may have become an important factor hindering the development in this field. More studies should be warranted to promote the use of tissue-engineered bladders in clinical practice.

## Introduction

The clinical manifestation of urinary incontinence or upper urinary tract impairment due to gradual decline in bladder function can be caused by congenital and acquired conditions (1) (such as bladder exstrophy, neurogenic bladder, and malignancies). Bladder augmentation is a feasible method to prevent renal impairment for patients with low compliance and/or high bladder pressure resulting in vesicoureteral reflux, hydronephrosis, and impaired upper urinary tract function when conservative treatment fails.

Currently, the augmentation or replacement of bladder with intestinal tissue is the gold standard method for end-stage neurogenic bladder with upper urinary tract damage (2). However, various complications, such as metabolic disturbance, mucus production, urolithiasis, infections (3), and even malignancy (4), are associated with bladder augmentation with intestinal tissue. For the purpose of avoiding complications and encouraging extensive surgical applications, there is an urgent need for alternative and innovative therapeutic approaches of tissue engineering.

**Figure 1:**
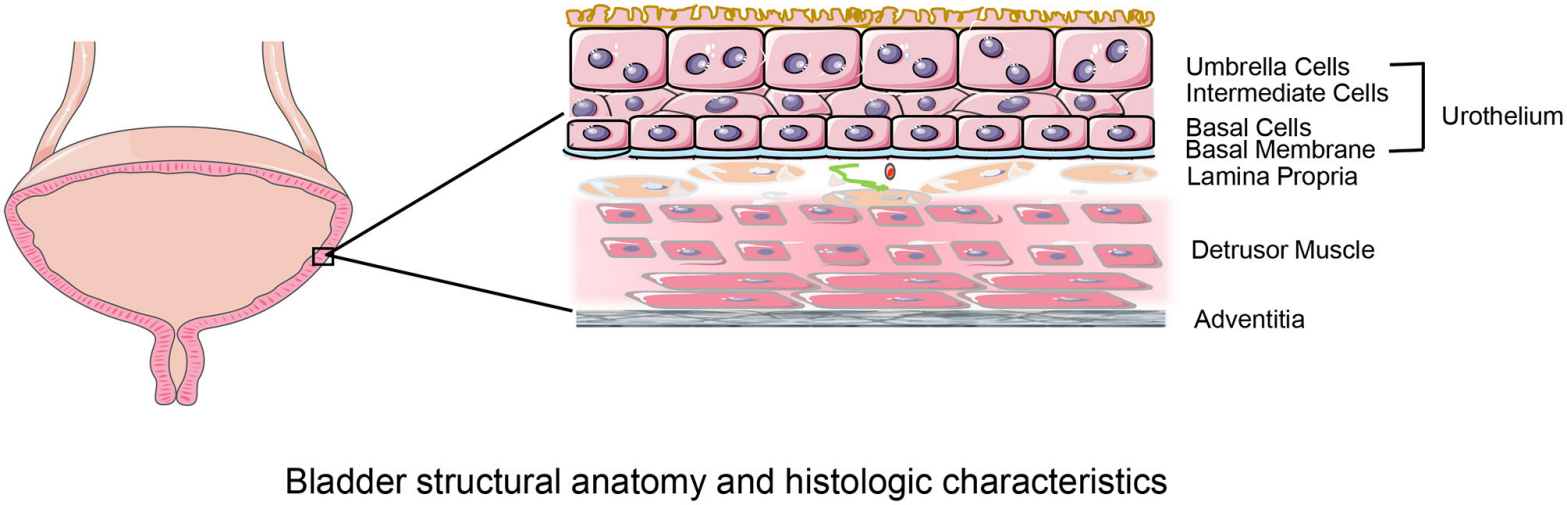
Bladder structural anatomy and histologic characteristics. The bladder walls consist of four layers: urothelium; lamina propria; muscular layer and serosal layer. The urothelium, composed of umbrella cells, intermediate cells, basal cells, basal membrane lines the bladder lumen and forms the urine-body barrier. The lamina propria is a connective tissue layer that contains nerves and vessels. The detrusor muscle layer consisted of longitudinal and transverse muscles that provides structural support to the bladder and facilitates its physiological functions of filling and emptying. The serosal layer covering the external surface is the outermost layer.

Over the last two decades, the accumulated knowledge of bladder histology and function (Figure 1) as well as the progress we made in tissue-engineering technology have promoted the development of bladder regeneration. Tissue-engineering technology for bladder augmentation or regeneration holds strong potential in patients with neurogenic bladder who developed renal impairment by decreasing surgical time and reducing complications compared with conventional enterocystoplasty. Tissue-engineering technology could provide novel treatment options for bladder augmentation by regenerating epithelium and muscle using a variety of biomaterial scaffolds, along with autologous, or allogeneic cells and growth factors. This approach might lead to the regeneration of partial bladder tissue or construction of a neo-bladder (5).

Despite that tissue engineering appears promising for bladder augmentation, there has been limited application in clinical settings. In this review, we discussed the current status of bioengineered bladder with particular emphasis on the biomaterials and cells being used. Furthermore, we highlighted the current problems of bioengineered bladders and suggested future research directions of bioengineering approaches for bladder augmentation and reconstruction.

## Methods

A literature search was performed in PubMed on December 1, 2020, with the following search terms used: (“bladder” [All Fields] AND “tissue engineering” [All Fields]) OR (“printing” [All Fields] AND “bladder” [All Fields]) OR (“3D printing” [All Fields]). Initial screening using titles and abstracts was performed to identify relevant studies. Selected records were further categorized into clinical studies, animal experiments, and review articles, in order to identify the status and problems of tissue-engineered bladder augmentation or reconstruction. We also proposed an innovative method of bioengineered bladder by combining the available tissue engineering technologies.

### Bladder Augmentation by Tissue Engineering

In recent years, tissue-engineering technology has been dedicated to the fabrication of functional bladder and has achieved remarkable achievements. Two main determinants of augmentation or reconstruction of bioengineered bladder include the growth and maturation of cells on the matrix and the formation of temporary or applicable scaffolds to achieve vesical functions (6). Scaffolds support cell growth and interaction, nutrient and oxygen transport, and metabolic wastes discharge. Besides, biomaterials can hold adequate mechanical properties at the preliminary stage of tissue engineering bladder augmentation and degrade at the later stage.

Acellular scaffolds and cellular scaffolds, two major methods of tissue engineering, are most widely used in experiments to induce bladder regeneration (7). Acellular scaffolds, composed of natural or synthetic biomaterials, can act as a temporary support for cells to activate spontaneous regenerative mechanism in bladder. Cellular scaffolds, including biomaterials and cells, correspond to the histology structure of bladder. Both cellular and acellular scaffolds *in vitro* and *in vivo* (8) have revealed excellent biocompatibility.

### (1) Natural Bladder Scaffolds

Natural acellular scaffolds are typically derived from bladder acellular matrix (BAM) (9) or small intestine submucosa (SIS) (10). The composition, microstructure, and mechanical properties of these materials are similar to the native tissues (11). Furthermore, these collagen-rich scaffolds maintain cell ingrowth and differentiation to accomplish the regeneration of bladder wall, and slowly degrade after the implantation without immunogenic rejection (12).

Up to now, the promising SIS scaffolds for urinary bladder augmentation have been widely applied in bladder augmentation both pre-clinically and clinically with different outcomes. The study of bladder augmentation in patients with exstrophy showed that acellular scaffolds acting as the temporary support could allow spontaneous regeneration of urothelial cells (UCs). However, smooth muscle cells (SMCs) (13), which were incapable of regeneration, could only be generated by a cell-based approach. Lu et al. (14) compared muscle-derived cells (MDCs)-SIS to SIS for tissue-engineered bladder reconstruction. The results indicated that MDCs migrated throughout SIS, which developed to muscle layers, and the areal strain of MDCs-SIS were significantly increased compared with SIS alone. The comparison of SIS acting as a 3D scaffold with and without SMCs and UCs were carried out by Zhang et al. (15). The study demonstrated that both groups presented moderate-to-heavy adhesion and shrinkage in grafts, calcification, and formation of bone. BAM, derived from bladder submucosa, is another great biomaterial for bladder regeneration. BAM was first applied to bladder regeneration *in vivo* rat model study in 1997 (16). The study results suggested that all bladder wall components involving UCs and SMCs facilitated BAM scaffold ingrowth and obtained normal bladder capacities. Coutu et al. (17) compared the outcomes of bladder replacement or augmentation using SMCs-seeded and SMCs-unseeded BAM and reported normal bladder capacity in SMCs-seeded BAM. Based on the results of existing literature, researchers believed that SIS and BAM implanted with cells, an ideal option for bladder augmentation, acquired better functional outcomes than scaffolds without cells in animal studies. Therefore, both materials were used in initial human bladder augmentations.

Naturally derived biodegradable materials, such as hyaluronic acid (HA) (18) and alginate (19), have also been used for bladder regeneration. However, infection, stone formation, rupture, and graft fibrosis caused by long-term invasion of urine have prevented their widespread clinical application. In the last 5 years, matrix self-assembled by mesenchymal cells (20) and anchored mesenchymal cell-seeded collagen gels (21) have been proved to improve the normal urothelial differentiation. However, these results need to be confirmed *in vivo*.

### (2) Synthetic Bladder Scaffolds

Bladder synthetic scaffold composed of polymers, which regulates the physical and chemical properties through material and synthetic reactions, is also one of the most hopeful bioengineering approaches to bladder regeneration. Synthetic scaffolds can provide better mechanical endurance and dimensional stability compared with biological scaffolds. Biocompatible, degradable, and non-toxic tissue engineering grafts have been certified by the Food and Drug Administration (FDA) for human subjects (22, 23).

It has been shown that the use of synthetic materials alone for urinary bladder regeneration was associated with a certain degree of complications, such as graft shrinkage, leakage urine (24), collapse, and cicatrization (24). Jayo et al. (25) reported that poly (lactic-co-glycolic acid) (PLGA) biodegradable scaffolds have been successfully seeded with UCs and SMCs for bladder reconstruction. This study clearly showed that tissue-engineered neo-bladder, which delivered autologous UCs and SMCs to biodegradable polymer, was able to approach or even exceed the pre-cystectomy bladder capacities at 6 months after transplantation. Furthermore, the compliance of neo-bladders was similar to the pre-cystectomy values, and a normal cellular organization, including a trilayer of urothelium, submucosa, and muscle, was reported by the bladder biopsy (25).

Other potential synthetic materials of bladder scaffolds include polyanhcydride, polyester, and silk fibroin (26). However, the lack of bioactive factors of natural biological tissue is one of the obvious disadvantages of synthetic bladder scaffolds. Bioactive factors play important roles in proliferation, migration, and differentiation of several types of cell (27). Furthermore, the acidic by-products produced by the degradation process may reduce the pH value around synthetic scaffolds and provoke minor foreign body rejection.

### (3) Composite Bladder Scaffolds

Composite biomaterials that combined the merits of at least two biomaterials with different properties have distinct advantages in many physical and chemical properties. Integrating the characteristics of biomaterials from different sources into composite bladder scaffolds not only revealed the limitation of applying one particular biomaterial, but also exploited the benefits of composite biomaterials.

Composite scaffolds with properties of natural acellular collagen matrix and PGA polymer have set the expectation in tissue engineering of hollow organs and tissues (28). The mechanical strength similar to the native bladder and the biological environment suitable for tissue growth were achieved through the creation of the hybrid construct. Acellular matrix, the inner side of bladder cavity supporting UCs ingrowth, acts as a barrier. On the outside, synthetic polymer, designed with large pores, accommodates SMCs and maintains the structure. It has been reported that plastic-compressed collagen-poly (lactic acid-co-ε-caprolactone) (PLAC) hybrids could be a possible material for engineered bladder scaffolds without inflammatory reaction, suggesting that SMCs and UCs proliferate well in composite bladder scaffolds (29). Recently, BAM coated with electrospun PLGA has been used as a unique biomaterial for tissue engineering (30). The PLGA material boosted the mechanical tension of BAM and reduced the shrinkage of grafts. Normal bladder capacity was maintained due to the mixture of two material properties.

Notably, composite scaffolds used for tissue-engineered bladder are in their early development. Composite scaffolds carrying several cell types seem to be a potential option for tissue engineering of hollow organs such as bladders. Future studies will focus on safety evaluations and efficacy assessments in animal models (31).

In addition, nanotechnology can transform the surface energy of implanted materials to alter initial protein adsorption events important for promoting tissue regeneration (32). Due to the small nanometer surface sizes as well as excellent biocompatibility properties, the study of bladder regeneration materials based on nanosurface features has become a hotspot.

### (4) Cells and Environment

As mentioned above, studies have demonstrated that cell-seeded scaffolds could activate self-regeneration of bladder tissue and were ideal for inducing urinary bladder wall regeneration compared to cell-free scaffolds. Currently, major approaches to tissue engineered bladder augmentation with implanted cells involve in the use of stem cells and urothelial cells.

**Figure 2:**
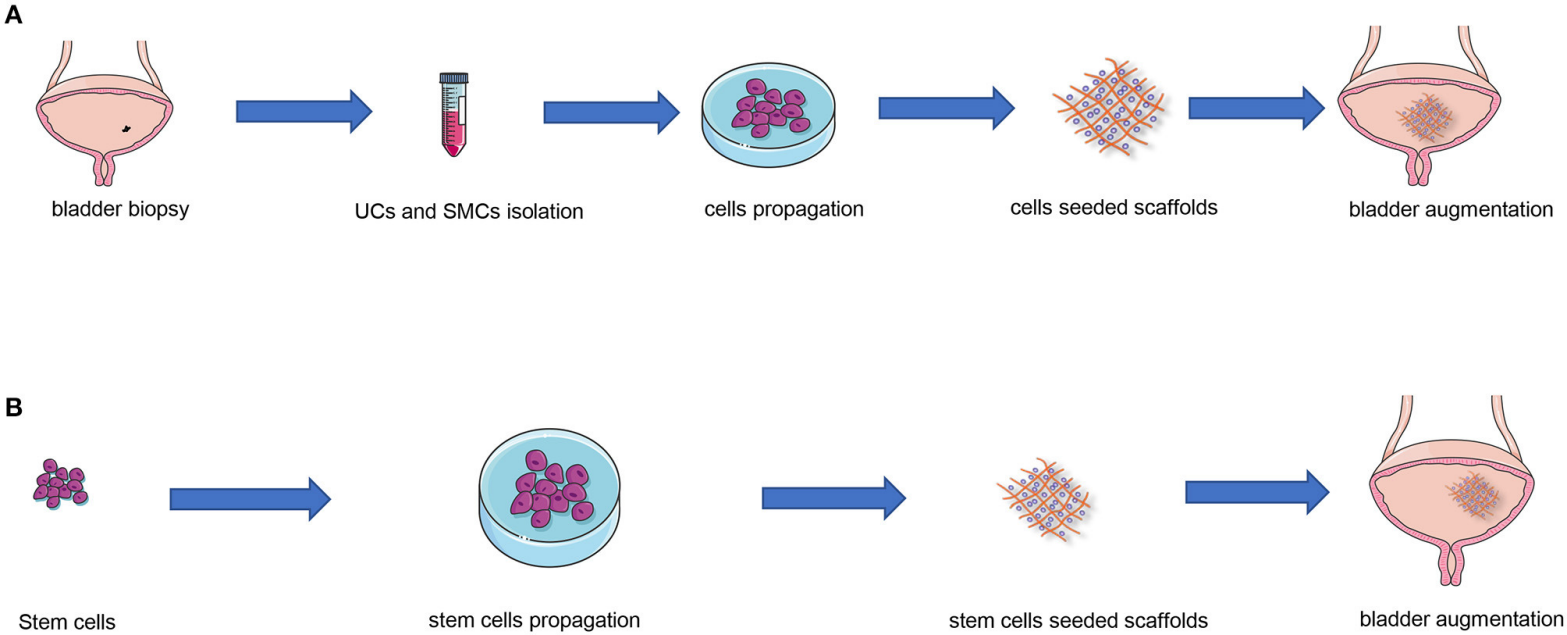
Tissue engineering strategies for autologous urothelial cells and stem cells. **(A)** UCs and SMCs obtained from the biopsy material first proliferate in the cell culture incubator; then the proliferated cells are subsequently reseeded into a tissue-engineered scaffold; eventually, the scaffold with cells was reimplanted into the same host. **(B)** Firstly, stem cells proliferate in the cell culture incubator; subsequently, the proliferated cells are seeded into a tissue-engineered scaffold; eventually, the scaffold with cells was implanted into the host. UCs, urothelial cells; SMCs, smooth muscle cells.

Autologous urothelial cell implantation is one of the ideal options for tissue engineering since it prevents inflammation and rejection associated with non-autologous tissue. As shown in Figure 2A, urothelial cells obtained from the biopsy material first proliferate in the cell culture incubator; the proliferated cells are subsequently reseeded into a tissue-engineered scaffold; eventually, the scaffold with cells was reimplanted into the same host (33). The first clinical study of tissue engineered autologous UCs and SMCs in patients needing cystoplasty was performed by Atala et al. (5). Over the last decades, several studies of urothelium seeded in various types of scaffolds, such as natural scaffolds (15), synthetic scaffolds (25), and composite scaffolds (34), have been conducted. However, tissue engineering seeded with cells derived from autologous bladder is probably not suitable for patients with cancer or neurogenic bladder. Cells from neurogenic bladder could alter genetic or pathologic phenotypes of the cultured grafts, including proliferation, adhesion, or decreased cell contractility (35). Moreover, the problem that UCs from urologic patients have a very few proliferative potential can not be ignored (36).

In addition to autologous urothelial cells, stem cells may be an alternative for tissue engineering bladder augmentation (Figure 2B). Mesenchymal stem cells (MSCs), originating from bone marrow or adipose tissue, have been shown to have positive effects on tissue engineering in experimental animals (37–39) and safety and feasibility in clinical practice (40). Additionally, induced pluripotent stem cells (iPSCs), which were generated artificially by reprogramming somatic cells, can differentiate into urothelial cells or smooth muscle cells under specific microenvironment (41). However, embryonic stem cells (ESCs), which are able to self-renew and differentiate into any type of cell, have been shown to transdifferentiate into teratomas, thus still remain intense medical and ethical controversies for human application (42).

Microenvironment plays an important role in proliferation, migration and differentiation of several cell types. Cells implanted on scaffolds are strongly impacted by different microenvironment factors, including extracellular matrix (ECM), growth factors, and chemical and physical stimuli (6). ECM with structural proteins as the main component acts as a repository for growth factors and other nutrients, in order to distribute bioactive components. Growth factors and nutrients promote the regeneration of bladder tissue, resulting in enhanced angiogenesis and better ingrowth of UCs and SMCs (43). Another novel approach to simulate the microenvironment of cell growth is to combine bioengineered structures with bioreactors *in vivo* or *in vitro*. Bioreactors can promote tissue maturation and enhance mechanical properties by controlling microenvironment, such as pH, temperature, oxygen concentration, and mechanical environment.

### (5) Bladder Tissue Engineering in Clinical Settings

In our review of clinical studies regarding tissue engineering for bladder regeneration to date, none of the results was satisfactory. The initial clinical trials of bladder reconstruction using plastic molds were considered as the prototype for tissue-engineered bladder research. However, the outcomes of clinical trials in the past decades indicated that plastic molds (44), gelatin sponges (45), preserved dog bladders (46), and lyophilized human dura (47) used in bladder reconstruction were associated with varying degrees of complications. The generated new-bladders have developed obvious fibrosis and underwent shrinkage over time (1). Other common complications were vesicoureteric reflux, upper urinary tract dilatation, recurrent infections, and urine leakage. As a result, the high rates of complications and mortality have led to the abandonment of these trials. Despite that gelatin sponges and Japanese paper sprayed with nobecutane have obtained satisfactory results in the initial phase of bladder augmentation, further clinical trials should be warranted in order to confirm their efficacy and safety (1).

From 2012 to 2014, several studies utilized porcine SIS for bladder augmentation. The pilot experience in 5 exstrophic patients showed that bladder capacity and compliance increased 30% at 6 months and remained stable at 18 months (48). The study of Liao for bladder augmentation reported there were modest increases in bladder capacity at 6 months of postoperative follow-up, and about 40% of the patients indicated low satisfaction for the new-bladder function (49, 50). In another Phase II study in children and adolescents with spina bifida, the results also showed that implantation of biodegradable scaffolds with autologous cells did not significantly improve the bladder compliance or capacity, and reported serious adverse events exceeding an acceptable safety criterion (51). In consequence, the long-term follow-up demonstrated that bladder capacity and compliance was poorly increased to obtain significant clinical benefit, and most of authors suggested that enterocystoplasty could not be substituted by SIS or biodegradable scaffolds (48–52).

### Challenges

The goal of tissue engineering is to create a bioengineered bladder as a substitute for the natural one *in vitro*. Tissue engineering applications in partial bladder reconstruction or augmentation seem to be a promising way. However, clinical applications of bladder tissue engineering have not been established currently. Clinical studies still face serious challenges due to technical limitations and unstable results. According to previous studies, several factors of tissue engineering used in bladder augmentation remain to be further resolved:

### (1) Mechanical Properties

The mechanical behavior of the graft, which is similar to the native one, is particularly important in bladder regeneration process (53). Tissue engineering technique used for bladder augmentation is designed to prevent progressive renal disease by increasing bladder volume, decreasing bladder pressure, and improving compliance and continence (50). The mechanical properties of bowel wall, the gold standard for bladder reconstruction, are considered to be the basis of ongoing research (13). Accordingly, creating a graft that can mimic the mechanical and functional properties of bladder wall becomes the core of tissue engineering. Current biotechnology struggles to explore an ideal biomaterial that can mimic the structure, biomechanics, and physiology of natural bladder.

### (2) Small Graft Size

Clinical applications of bladder augmentation with tissue-engineering technology have not been determined due to the inconsistent functional results of bladder grafts. Atala et al. (5) and Zhang and Liao (49), used tissue-engineered grafts of 70–150 cm^2^, reported positive outcomes. Graft size in other studies was either smaller or missing. Five-year follow-up of the above studies showed an average increase of 275.6 (275.6 ± 159.5) ml in maximum bladder volume, but not enough to mitigate the progression of upper urinary tract disease (50). Although a larger area of tissue-engineered grafts for bladder augmentation may yield satisfactory results, for a large bladder graft, extensive cell regeneration occurs in the peripheral area of the graft, while the center is lack of organized smooth muscle bundles and urinary epithelium (54). In a rabbit bladder augmentation model with 70% partial cystectomy, neither normal bladder capacity nor organized smooth muscle bundles was restored by SIS with or without autologous SMC seeding (55). The efficacy of large tissue-engineered grafts for bladder augmentation surgery remains to be further explored.

### (3) Vascularization

Rapid neovascularization is essential for graft survival and organ structure and function. However, a major obstacle for tissue engineering lies in the formation of post-implantation vascular network being capable of perfusing the regenerated tissue (56). Similarly, one of the major barriers for large graft survival is vascularization. Tissue regeneration may be hampered by deficient oxygen and nutrition and inadequate removal of waste products, leading to the loss of bladder function and necrosis. It was reported that spontaneous angiogenesis, omental coverage, or application of exogenous angiogenic factors could enhance capillary growth of the graft, but these processes were still not reliable to maintain the blood supply of large grafts (7). In order to overcome this obstacle, one of the available choices is to use 3D bio-printing technology to design and generate vascular network (57, 58). In addition, it is also a promising option to use the reseeded stroma technology to form a 3D capillary-like network (59).

### (4) Fibrotic Reaction of the Graft

Implantation of bladder scaffolds with different biomaterials typically triggers fibrotic reaction of the graft to varying degrees *in vivo* (60). Urine was considered to be one of hazard factors made the urinary bladder unfavorable for induced regeneration. In the early stages of urothelium regeneration when the epithelial protective barrier was dysfunctional, urine developed a deleterious effect on the cellular components of the tissue-engineered bladder. Local fibrosis primarily occurred in urinary bladder and gradually develop to abdomen, eventually causing abdominal adhesion or even mechanical obstruction and intestinal necrosis. Thus, suppressing fibrotic reaction and transplant rejection of the bioengineered new-bladder are major tasks. Previous studies have also demonstrated that it was necessary to synergistically inhibit multiple pro-fibrotic cascade reactions (61, 62). According to current results, cells and anti-fibrotic agents incorporated within the scaffold would be effective options to overcome graft with fibrosis (7, 13). Beside, urine-derived stem cells, which are more resistant to urine than other types of cells, may be an ideal candidate for tissue-engineered bladders (63).

### (5) Innervation

Restoration of the bioengineered new-bladder innervation is one of the most challenging issues (64). Innervation of the bioengineered neobladder is essential for graft function (normal urine storage and urination) and long-term survival. It should be noted, however, that patients with neurogenic bladder undergo bladder augmentation, in order to expand urinary bladder capacity and decrease bladder pressure. Patients can maintain the remaining renal function by emptying their bladder regularly with intermittent catheterization (65). In this context, it is necessary to maintain normal shape and sufficient volume of tissue-engineered bladder, without reconstructing the innervation of urinary bladder.

### Future Prospects

Bioprinting is an encouraging technology for organ and tissue manufacturing, compared with other tissue engineering techniques, because it theoretically allows the close mimic of the anatomic structure. Several factors may predict the expected success of 3D bioprinting as follows:

(1) Compared with traditional bioengineered manufacturing methods, 3D bio-printing can provide structures with scaffold microstructures and cell arrangements that have been prepared by designers. The scaffold offers a more suitable growth environment for seed cells by strengthening cells contact and cell–cell and cell–matrix interaction (66); (2) Bioprinting scaffolds possess suitable pore size, porosity, and interpore connectivity (67), which is conducive to adhesion, growth, and differentiation of seed cells; (3) Bioprinting technology is likely to resolve the problem of angiogenesis in bioengineered tissues. Ten years ago, the application of improved thermal inkjet printer has demonstrated the ability to print capillaries (68). Generating a network with hollow structure with sacrificial filaments becomes another feasible method (69), which can be obtained by injecting vascular endothelial cells into the hollow network. Up to now, there are many types of tissue and organ, apart from cardiac tissue and liver, which are trying to vascularize.

Currently, the major trend of clinical treatments includes minimally invasive and natural orifice transluminal endoscopic surgery. In recent years, *in situ* bioprinting technology as a hot spot has received worldwide attention (70). Albanna et al. (71) reported that printing autogenous skin cells into skin wounds could speed up the healing of large wounds. The concept of living bioprinting *in situ in vivo* proposed by Xu et al. (72) makes up for the shortcomings of traditional biological printing technology. The miniature 3D bioprinting device they developed, which is similar to a gastroscope, can be mounted on the endoscope and perform *in situ* inkjet printing at the injured spot of stomach wall after being inserted into gastral cavity. This technique provides a new idea for the diagnosis and treatment of gastric wall injury. *In situ* bioprinting may have several advantages: (1) Compared with bioprinting *in vitro*, this bioprinting approach may be more compatible with the microenvironment in which the seed cells grow (70). Suitable microenvironment *in vivo* is beneficial to cell adhesion, proliferation and differentiation; (2) *In situ in vivo* bioprinting may obviate the necessity of bioreactors, a group of sophisticated engineering simulation biosystems. The peritoneal cavity and bladder wall, as the bioreactors to create a natural environment for cellular growth and differentiation, can also prevent graft inflammation and fibrosis caused by ischemic injury, as the graft is transferred from the bioreactor system to the surgical location (73, 74).

**Figure 3:**
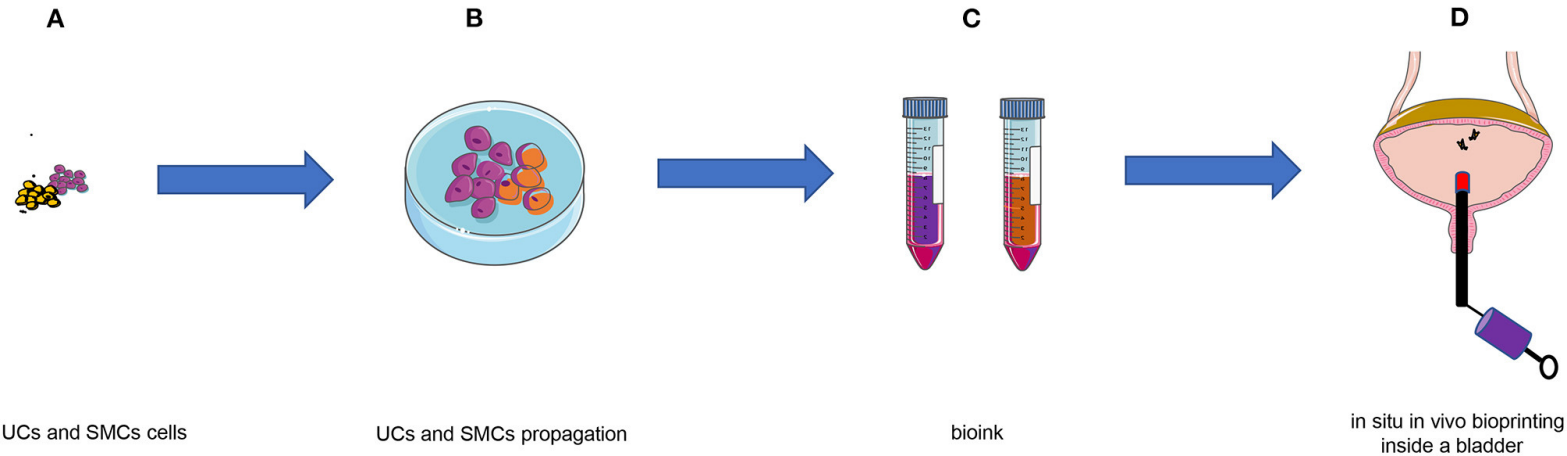
Schematic of *in situ in vivo* bioprinting taking the case of bladder augmentation. **(A)** UCs and SMCs were harvested from biopsy material; **(B)** UCs and SMCs proliferated *in vitro*; **(C)** Bioinks containing UCs and SMCs; **(D)** *in situ in vivo* bioprinting after bladder augmentation with biomaterial. UCs, urothelial cells; SMCs, smooth muscle cells.

**Figure 4:**
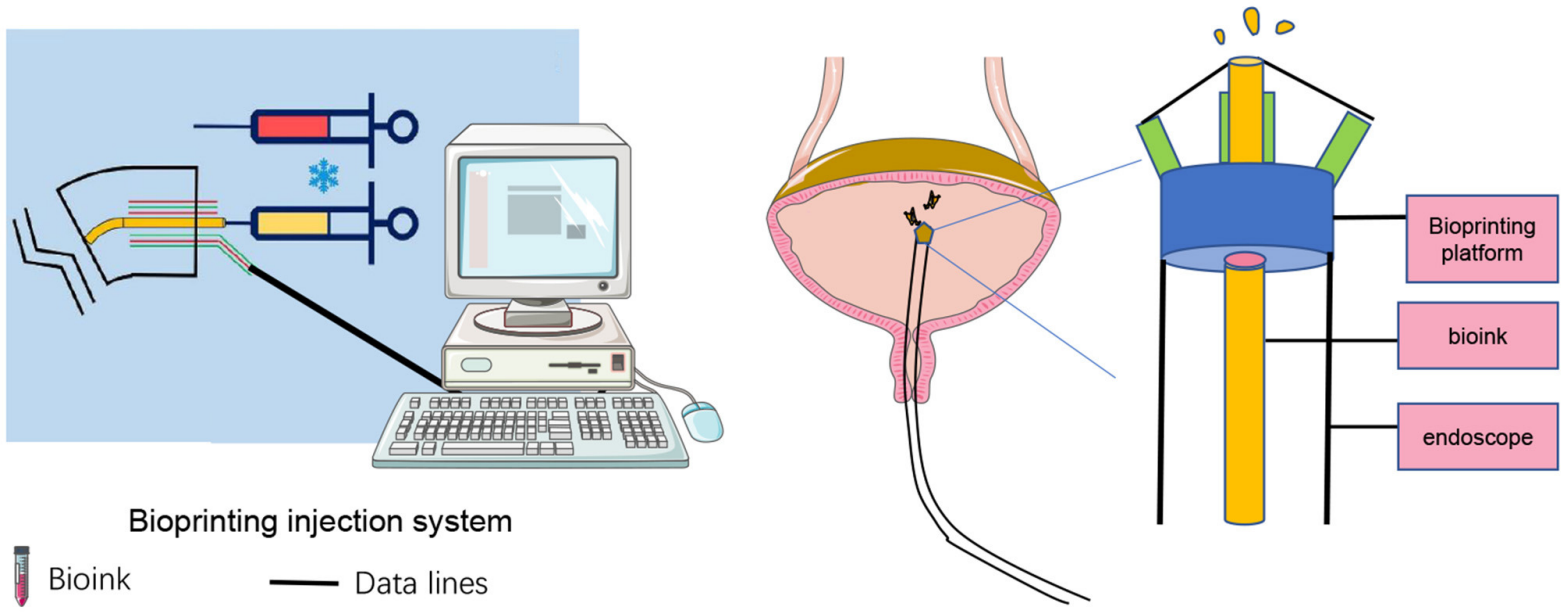
Schematic of *in situ in vivo* bioprinting inside a bladder.

Based on the above benefits, the combination of 3D bioprinting technology and *in situ in vivo* bioprinting may be the main research direction in the future. This emerging technology would undoubtedly make substantial progress in tissue engineering for clinical application (72). Inspired by the study of bioprinting *in situ* at the gastric wound site, we hypothesize that micro-intravesical 3D bioprinting is a promising option for bladder augmentation surgery. After screening for the appropriate biomaterial of scaffolds for bladder augmentation, a novel micro bioprinting platform will be used for *in situ in vivo* bioprinting at the implant location through urethra (Figures 3, 4). This approach takes the advantage of internal environment, with the pelvic cavity serving as a natural bioreactor to promote angiogenesis and reduce fibrotic reaction (73). Moreover, we consider that transurethral bioprinting can print repeatedly to repair the damaged and detached cells, thereby promoting the growth of new bladder and reducing the associated complications, such as atrophy, perforation, and rupture.

## Conclusion

Tissue engineered grafts aimed to substitute enterocystoplasty will become in future new gold standard of reconstructive urology. However, the application of tissue-engineered bladder augementation in clinical practice remains a great challenge. This work presents an innovative method for tissue engineering of bladder augmentation, but the conclusions of this scheme remain to be further confirmed. We are confident that, step by step, bioprinting will eventually be an effective clinical protocol for bladder amplification in the near future.

## Author Contributions

LL conceived the idea for the manuscript. XW drafted the manuscript with significant contribution from LL. XW and FZ revised the manuscript. All authors contributed to the article and approved the submitted version.

## Conflict of Interest

The authors declare that the research was conducted in the absence of any commercial or financial relationships that could be construed as a potential conflict of interest. The reviewer PZ declared a shared affiliation, with no collaboration, with the authors to the handling editor at the time of the review.
